# The Transporter-Mediated Cellular Uptake and Efflux of Pharmaceutical Drugs and Biotechnology Products: How and Why Phospholipid Bilayer Transport Is Negligible in Real Biomembranes

**DOI:** 10.3390/molecules26185629

**Published:** 2021-09-16

**Authors:** Douglas B. Kell

**Affiliations:** 1 Department of Biochemistry and Systems Biology, Institute of Systems, Molecular and Integrative Biology, University of Liverpool, Crown St, Liverpool L69 7ZB, UK; dbk@liv.ac.uk; 2 Novo Nordisk Foundation Centre for Biosustainability, Technical University of Denmark, Building 220, Kemitorvet, 2800 Kgs Lyngby, Denmark; 3 Mellizyme Biotechnology Ltd., IC1, Liverpool Science Park, Mount Pleasant, Liverpool L3 5TF, UK

**Keywords:** membrane transport, pharmaceuticals, drugs, energy coupling, biotechnology, ADME, DMPK, transporter engineering

## Abstract

Over the years, my colleagues and I have come to realise that the likelihood of pharmaceutical drugs being able to diffuse through whatever unhindered phospholipid bilayer may exist in intact biological membranes in vivo is vanishingly low. This is because (i) most real biomembranes are mostly protein, not lipid, (ii) unlike purely lipid bilayers that can form transient aqueous channels, the high concentrations of proteins serve to stop such activity, (iii) natural evolution long ago selected against transport methods that just let any undesirable products enter a cell, (iv) transporters have now been identified for all kinds of molecules (even water) that were once thought not to require them, (v) many experiments show a massive variation in the uptake of drugs between different cells, tissues, and organisms, that cannot be explained if lipid bilayer transport is significant or if efflux were the only differentiator, and (vi) many experiments that manipulate the expression level of individual transporters as an independent variable demonstrate their role in drug and nutrient uptake (including in cytotoxicity or adverse drug reactions). This makes such transporters valuable both as a means of targeting drugs (not least anti-infectives) to selected cells or tissues and also as drug targets. The same considerations apply to the exploitation of substrate uptake and product efflux transporters in biotechnology. We are also beginning to recognise that transporters are more promiscuous, and antiporter activity is much more widespread, than had been realised, and that such processes are adaptive (i.e., were selected by natural evolution). The purpose of the present review is to summarise the above, and to rehearse and update readers on recent developments. These developments lead us to retain and indeed to strengthen our contention that for transmembrane pharmaceutical drug transport “phospholipid bilayer transport is negligible”.

## 1. Introduction

**Figure 1:**
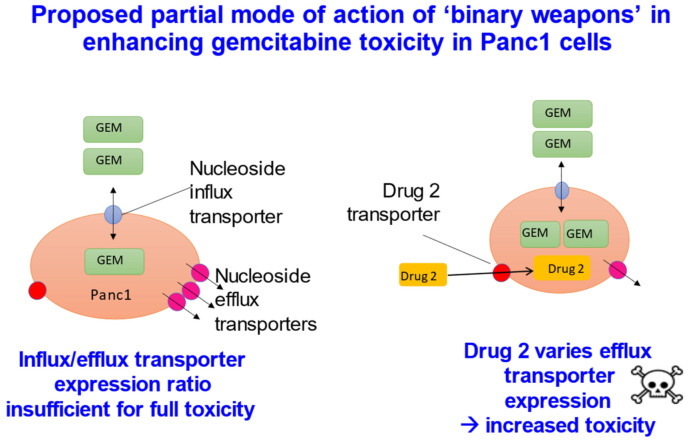
A drug D might pass through a biological membrane in one of two main ways conceptually (we do not here discuss endo- and exocytosis; the focus is only on cases where the drug is considered to cross through the membrane barrier itself). On the left is illustrated transport through phospholipid bilayers, while on the right we illustrate the use of proteinaceous solute carriers to effect entry and exit of the drug. The crux of this review is that the mode on the left does not take place at any meaningful rate in intact biological membranes (since they have a high protein content).

Over the years, two main ideas have been used to explain the mechanisms by which pharmaceutical drugs and/or substrates and products of biotechnological interest pass through the plasma (or other) membranes of the relevant organism: in a more classical analysis, it is assumed (and it really is purely an assumption [1]) that molecules can diffuse through the core of the lipid bilayer by some means. In an alternative and really entirely opposite view, which we refer to as PBIN for “phospholipid bilayer diffusion is negligible” [2], we have argued [1,2,3,4,5,6,7,8,9,10,11,12] (and these constitute background that we mainly do not rehearse again here) that this does not in fact occur in real biomembranes to any significant extent at all. Some of this background material is also available in a webinar (https://bit.ly/3yQJ1FG, accessed on 15 September 2021). The two modes are illustrated in Figure 1. One may debate what is “significant” and/or “negligible”, but anything less than 5% of a total flux really is not much of a contribution, and plenty of evidence implies that it is almost certainly less than 1%. In our view, the term “negligible” covers this more than adequately. The purpose of this review and commentary is to provide an update on some of the salient issues. Examples of the importance of transporters continue to grow apace, while—whatever may or may not happen in artificial membranes—it remains the case that the actual evidence for significant transmembrane transport solely through any bilayer portions of intact biological membranes is non-existent. We shall start by looking at why this is the case, initially by focusing on some of the major differences between real biomembranes and those artificial ones made from phospholipid bilayers.

### 1.1. Biological Membrane Structure

**Figure 2:**
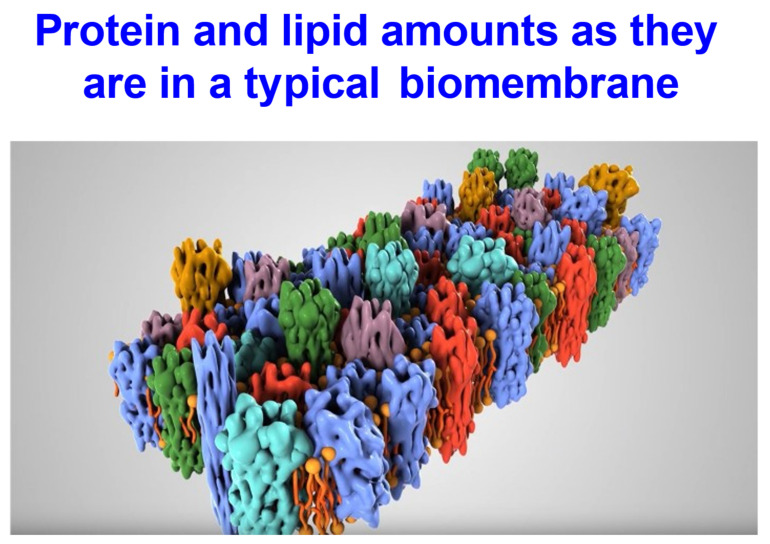
Cartoon of a typical biomembrane indicating the relative paucity of phopholipid bilayer that is uninfluenced by proteins. Taken from an Open Access animation covering some of this ground and related transporter matters at https://www.youtube.com/watch?v=s23vNwLE-Jw.

**Figure 3:**
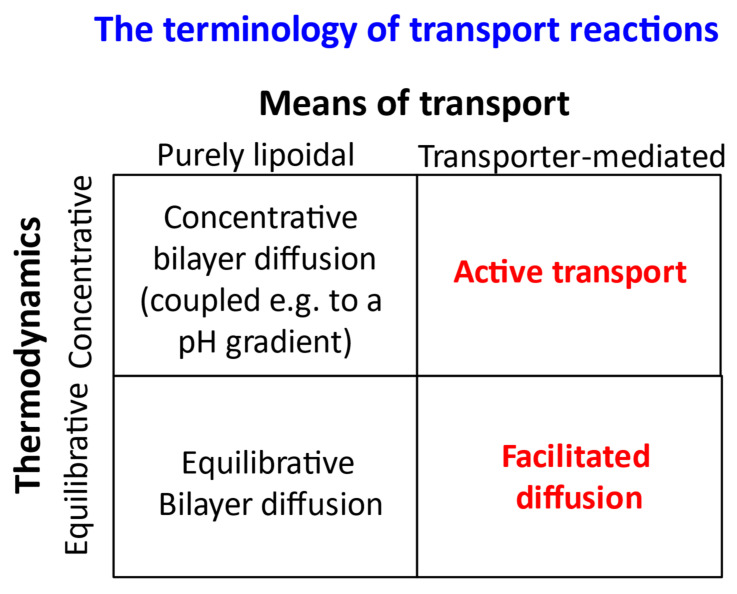
Suggested terminologies to avoid the use of the term “passive”, which is still widely misused to conflate two entirely separate concepts, one thermodynamic (hence independent of mechanism) and one mechanistic.

The textbook view of biological membranes is that they are to be seen as a “fluid mosaic” [13,14] of proteins embedded within a phospholipid bilayer, along with other small molecules such as sterols. The original article [13] featured a now iconic illustration (shown in Figure 3 of said article) in which a small number of proteins were embedded in and on a “sea” of phospholipids, with the proteins representing (by eye) approximately one-seventh of both the total mass and area. Unfortunately, this picture is very misleading, since biological membranes are commonly 3:1 protein:lipid by mass, and maybe 1:1 by area (**not** 1:7) [15,16,17,18,19,20,21,22,23,24,25,26,27,28] (see Figure 2). This widespread but erroneous mental picture, of “proteins floating in a sea of phospholipids”, has led many to suppose that from a biophysical point of view biomembranes are thus essentially just like pure lipid bilayers, in that the supposedly sparse proteins would do little or nothing to affect the kinds of properties that can be seen in purely phospholipid bilayers such as those studied in vitro [29,30,31]. However, this is emphatically not the case.

Purely artificial (solely phospholipid) bilayer membranes are not terribly stable and admit the passage of small molecules (and even ions) via transient aqueous pores or channels (e.g., [32,33,34,35,36,37,38,39,40,41,42,43]). This mode of transfer clearly has to be the case if ostensibly ‘transmembrane’ transport ‘through’ them is observed, as it can easily be calculated that the free energy necessary for passing a small cation through a dielectric (such as that represented by a membrane interior of phospholipid tails) with a permittivity of ca 2 is so great that it would be unlikely to occur even over millions of years [44]. More specifically, the lateral flexing of phospholipids needed to make such transient aqueous pores simply cannot occur when the phospholipids are either bound directly to a protein or are strongly influenced thereby (as they are, e.g., [45,46,47,48,49,50,51,52,53,54,55,56,57,58,59,60,61,62]). This is likely reinforced by the well-established, substantial and dynamic lipid asymmetry between the inner and outer halves of membranes [63,64,65]. Note too that ABC transporters are responsible for moving lipids themselves around cells and within membranes [47,66,67,68,69]. In short, studying an aardvaark tells you about an aardvark, not a langoustine. In a similar way, studying sodium chloride tells you about sodium chloride, not strychnine chloride, even if they both contain chloride. Thus, in a similar way, studying pure phospholipid bilayers tells you about pure phospholipid bilayers, and not biomembranes that may happen to contain relatively small amounts phospholipids in a bilayer form. This may seem obvious when set out in this way, which is why we do it.

### 1.2. Transport of Drugs through Biological Membranes and the (Mis)Use of the Term “Passive”

It is now well-established that cells require proteinaceous transporters to effect the transmembrane transport of the nutrients they need to survive and to grow. Since these nutrients are mainly small molecules, it might be thought that a similar degree of acceptance would accord to the assumption that this held true for pharmaceutical drugs too, as well as for the uptake and efflux of substances of interest to the biotechnology industry. Surprisingly, as mooted above, and for some comparable reasons with bioenergetics more generally [70], this has not largely been the case. Indeed, why these and other beliefs persist despite the facts, and what to do about it, is of general philosophical and psychological interest [71,72,73,74,75,76]. In this case, it seems from our experience that it is to do with culture and education; those versed in physical organic chemistry (who are often predominant in the DMPK/ADMET sections of pharmaceutical companies but also in more traditional pharmacology schools in academia) tend not to know much about enzymology, while molecular biologists—who tend to know little of physical organic chemistry—express extreme surprise when told of the widespread belief of physical organic chemists that drugs simply pass through the bilayer portions of biomembranes. There is clearly value for individuals from both backgrounds in learning a little of each topic.

The term “passive”, as employed for instance in the phrase “passive diffusion”, is also widely misused, not least because it can be (and is) used to cover and then conflate two separate concepts. The first represents a thermodynamic meaning, where “passive” is used to mean “equilibrative”: the transporter requires no free energy and simply lets molecules pass down their concentration gradients until their concentrations (strictly, thermodynamic activities) are the same on each side of the membrane of interest (i.e., that in which the transporter is embedded). Clearly, the thermodynamic usage can (or should) have no mechanistic implications. The problem is that the same term (as in “passive diffusion” or “passive permeability”) is also used to imply a mechanism, viz that such equilibrative diffusion occurs through the phospholipid bilayer. In the worst cases, demonstration of the thermodynamic property is used to imply or even to claim the demonstration of a mechanism as transbilayer diffusion. In our view, the term “passive” has acquired so much baggage that the only solution to this (Figure 3 and reference [2]), in addition to education, is to avoid the term “passive” completely, and thus be forced to be more explicit about precisely what it is that is being claimed in any particular case.

One feature of transport across membrane systems such as those of the popular Caco-2 cell monolayers [77,78,79,80,81,82,83,84], that (like other human tissues [85,86]; https://www.proteinatlas.org/search/slc), express hundreds of transporter proteins [87,88,89,90,91], is that the initial rate of transport of a given substrate at the same initial concentration from side A → side B can differ from that when the test is made from side B → side A, even when the transport is purely equilibrative. This has no ready explanation by or for those schooled purely in physico-chemical concepts and who believe that diffusion explains everything. By contrast, enzymologists have a perfectly straightforward explanation of this, which comes from the well-known thermodynamic Haldane relationship relating the K_m_ and V_max_ values in the forward (K_m,f_ and V_max,f_) and reverse (K_m,r_ and V_max,r_) directions of a reaction to its equilibrium constant K_eq_. While this can be found in any textbook of enzyme kinetics (e.g., [92,93,94]), or even of biochemistry, we reproduce it below:

(V_max,f_ · K_m,r_)/(V_max,r_ · K_m,f_) = K_eq_ (1)

Thus, even for K_eq_ values of unity, it is easily possible to have rates that differ manyfold in the two directions for the same external concentration, just by manipulating the other values in Equation (1) while keeping their ratios consistent with it. Of course, and I stress this purposely, this is true only for enzyme- (transporter)-mediated transport, not “diffusion” through lipid bilayers [5].

### 1.3. Untestability of Bilayer Transport Models in Real Biomembranes

Perhaps surprisingly, the view that molecules pass through phospholipid bilayers in real biological membranes is presently untestable, because we do not have the ability to image small molecules and membranes with the atomic resolution that would be necessary to observe their transport directly. What is presently carried out most commonly is to observe molecules on one side of a membrane and later on the other side, and simply assume that they appeared there via transbilayer transport. This is a well-established and classical logical fallacy known as “affirming the precedent” or “post hoc ergo propter hoc” [1]. In a different vein, observing a molecule transferring from one side of a pure phospholipid bilayer simply does not tell you what it might do in a real membrane where there is very little bilayer that is not affected by the presence of protein. Similarly, changing the type or amount of phospholipid does not “simply” do that, because the activities of membrane proteins, including transporters [95], can vary quite substantially with changes in their adjacent lipids. Consequently, any changes in transport induced by changing lipids can perfectly well (and more accurately) be explained by their influences on the proteins embedded in the membrane.

### 1.4. Testability of Transporter-Mediated Models in Real Biomembranes

**Figure 4:**
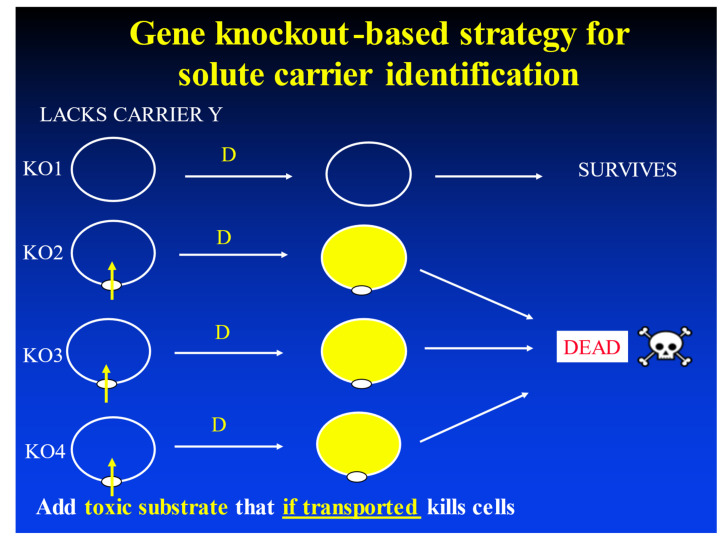
Principle of determining the substrate of a drug at toxic concentrations (e.g., [8,96,97,101,102]) by assessing the ability of cells lacking a particular transporter gene to survive its presence, while the wild-type cells, or cells knocked out for other genes not involved in the drug’s transport, are killed. Obviously, this is the extreme; there may also be degrees of resistance.

By contrast, those of us who claim that bilayer transport is negligible in real biomembranes can easily change the expression levels or activities of transporters of interest (e.g., [96,97]) and observe the concomitant effects on the transport of the small molecules of interest (as in Figure 4). (The same strategy can also be used to detect inhibitors of the transporter, e.g., [98,99].) In the paper of Winter et al. [97], the uptake and toxicity of an anticancer compound called YM155 or sepantronium bromide was decreased some 500-fold when the single transporter SLC35F2 was knocked out, and the toxicity of the compound correlated closely with that transporter’s expression level over some four orders of magnitude when explored in some 15 separate cell lines (that, as for many transporters [86,100], showed equivalently massively varying expression levels). The only reasonable (one might say plausible) explanation is that the SLC35F2 transporter normally carried some 99.5% of the flux of YM155 into the cell, and that consequently the transmembrane flux occurring by any other means, including via bilayer transport, is indeed negligible. This simple phenomenon served to explain entirely the highly variable efficacy of YM155 in a series of clinical trials.

Typically, the question of the interaction between substrates and transporters can be phrased in two complementary ways: the transporter-centric question is “given a transporter of interest X, what are its substrates?”. This is the problem of de-orphanisation [103,104,105,106,107,108,109]. The complementary, substrate-centric, question is “given a substrate Y, which transporter(s) is/are responsible for its cellular uptake and/or efflux?”. The latter is the more important one for assessing the mechanisms of drug transport [8], and there are many examples (e.g., [110,111,112,113,114,115,116,117,118]) where transporter activity has been identified but where the transporters involved have not. Of course, while a known result is a partial answer to each question, the strategies for tackling them are slightly different (for a recent overview of cell-based assay methods for SLCs, see [119]). Note, in particular, that simple biophysics plus the re-use of protein motifs in evolution means that most small molecules bind to multiple targets, and most proteins can bind small molecules promiscuously (e.g., [8,9,120,121,122,123,124,125,126,127,128,129,130]).

As part of the EU-IMI ReSolute project [131] (https://re-solute.eu/), the CEMM group studied 60 cytotoxic compounds. By using an SLC-focused CRISPR-Cas9 library, they identified a series of transporters whose absence induced resistance to the drugs tested [101], much as had been achieved in smaller numbers before [96,97]. This kind of substrate-centric strategy clearly represents a potent means of identifying drug transporters, and notably it also illuminated cases of interactions. Such interactions scale exponentially with the number of candidates, but high-throughput CRISPR-Cas methods are enabling the identification of all kinds of genes involved in complex biological processes (e.g., [132,133,134,135,136,137]).

### 1.5. Heterogeneity of Transport and Transporters in Different Cells and Tissues

If trans-bilayer permeability were significant in real biomembranes, the free concentrations of drugs inside cells and organelles (modulo pH and potential gradients) would be more or less homogeneous. Of course, they are not, as is well-known (and as can easily be determined by chemical imaging methods, e.g., [138,139,140,141,142,143,144,145,146,147,148,149,150,151,152,153,154,155,156,157,158,159]). A particularly well-established case is that of the blood–brain barrier, where, despite the lipids not being noticeably different from those in other cells, the permeability is negligible if specific transporters are not involved [160,161,162,163,164]. What does differ greatly between different cells and tissues, of course, is the expression of particular proteins such as transporter proteins [85,86,165], with any number of large datasets now becoming available.

### 1.6. Role of Transporters in Biotechnology

In addition to activities mentioned in our previous reviews that were focused on transporters and biotechnology [4,12,166], a number of other authors (as reviewed, e.g., in [167,168,169,170,171,172,173,174,175,176,177,178]) have also highlighted the importance of transporters in the uptake of CO_2_ [179] and in the biotechnological production of substances of interest in the BioEconomy. Some recent examples include the production of amorphadiene [180], citrate [181], L-malate [182,183,184], antibiotics [185], fatty acids [186], fatty alcohols [187], olefins [188], various organic acids [176], other dicarboxylic acids [189,190], sophorolipids [173,186,191,192], in microbial fluorination (by removing a fluoride effluxer from *E. coli* [193]), and in the production of a variety of hydrophobic substances [191,194]. Promiscuity can be quite significant for biotechnology [182,195,196,197,198,199]. In particular, here, the promiscuity of some transporters, especially under biotechnological conditions of unphysiologically high intracellular concentrations of small molecules, means that cells can have a tendency to leak pathway intermediates with structural similarities (as judged by various means [200,201]) to products, rather than simply just excreting the desired product; this too can be manipulated via transporter engineering [171].

### 1.7. Adaptive Laboratory Evolution and Membrane Transporters

**Figure 5:**
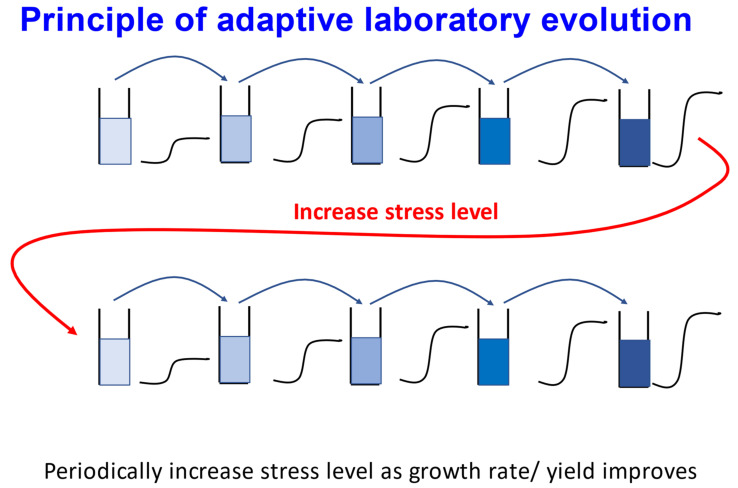
Illustration of the principle of adaptive laboratory evolution (ALE). Cells are exposed to a toxic substance that causes them to grow sub-optimally. Selection leads to strains that can revert to rates and extents of growth shown by the wild type when inoculated into fresh cultures. The stress level is increased and the process continued. Sampling and growth rate measurement can be completely automated.

While some studies are more binary, looking for resistance to a toxic substrate via survival or death “in one go”, other strategies (such as the very elegant “variable dose analysis” [202]) are more graded. A particularly nice example is that of adaptive laboratory evolution (ALE), illustrated in Figure 5. While many very elegant examples of long-term laboratory-based bacterial evolution exist (e.g., [203,204,205,206,207,208,209,210]), a more directed focus has been where it is exploited for biotechnology (e.g., [190,211,212,213,214,215,216,217,218,219,220,221,222,223]). However, it is perfectly applicable in drug transporter studies too. As with any phenotypic selection of this type [224,225,226,227,228,229,230,231], it relies on the fact that, in a heterogeneous population, the faster-growing strains will tend to become more prevalent at the expense of the slower-growing ones. Sequencing those that take over indicates where favourable mutational events have occurred, and essentially identifies the relevant genes. It can best be run as a hypothesis-free strategy [232], given that some mutations cannot reasonably be predicted (e.g., the role of ribosomal subunits in improving methanol tolerance in *E. coli* [233]). In one example involving transporters [234], ALE was used to increase the tolerance to aromatic amino acids of baker’s yeast. Here, [234], the major mutation was in a transcriptional activator called Aro80, that served to increase the activity of an efflux transporter Espb6, a role (in effluxing aromatic compounds) shared with the previously known Pdr12. In another example [235], *E. coli* cells were made more tolerant to ionic liquids; in this case, the chief mutations in tolerant clones occurred in transport processes in the shape of mdtJI, a multidrug efflux pump, and yhdP, a largely uncharacterised transporter possibly involved [236,237] in phospholipid transport to the outer membrane.

As illustrated in Figure 5, the commonest means of performing ALE is in a “semi-batch” type of mode in which small inocula are used to grow cells in batch mode, while they are then sampled at the end of growth and a new inoculum introduced to a separate batch culture. What is then selected, in part, is cells that as well as having a higher growth rate also have lowered lag phases and the ability to survive better in stationary phase. Truly continuous cultures provide for a much more stringent selection, especially when carried out in a turbidostat. In a turbidostat (e.g., [227,238,239,240,241,242,243,244,245]), the growth medium is arranged such that—unlike in a chemostat—cells can grow at the maximum rate they are able to within it. The biomass in a fermentor of working volume V is controlled at a set point via a suitable probe (see, e.g., [246,247,248]). As it exceeds this level, fresh growth medium (including any inhibitors) is pumped in at an average rate over a period of v mL · min^−1^. Cells are washed out at the same rate. As usual [249], this rate is numerically equivalent to the growth rate = the dilution rate = v/V min^−1^, and may be recorded continuously. Rather surprisingly, the method has not been widely used, although McGeachy et al. [227] give a nice example that illustrated selection of mutations in the Mep3p ammonium transporter. We predict that these kinds of strategies may have much more impact in the future.

### 1.8. Substrate Misannotations and the Importance of Antiporter Activity in Drug Influx and Efflux

Most transporters are discovered via their effects on the uptake or efflux of a particular substrate of interest, and they are often codified accordingly. Our experience is that—just as with enzyme annotation more generally [250]—this leads to misannotation in that “any” activity discovered first is typically seen the main or even only activity. A classic example is SLC22A4, previously known as OCTN1. It had been found to catalyse the uptake of carnitine, and also that of the the non-physiological tetraethylammonium cation. However, the rates were in fact quite miserable, and it was not until Gründemann and colleagues used what was effectively an untargeted metabolomics approach [103] that it was discovered that it was in fact “really” a concentrative, sodium-coupled transporter for the nutraceutical ergothioneine [251,252] and also for the related stachydrine (proline betaine) [103,253,254]. Equivalently, this example also serves to illustrate how finding a transporter with one activity does not mean that it is the only such capability, and it is now known that at least one more transporter, viz SLC22A15 [109], can also catalyse ergothioneine uptake.

Another means of misannotation is that based on an assumption of unidirectionality (as an “influxer” or an “effluxer”) that is not warranted and follows from the fact that most kinetic assays are set up to measure either only an influx or an efflux. At one level, bidirectional transport is inevitably the case, in that most equilibrative transporters are necessarily functionally perfectly reversible; if a transporter has two uniported substrates A and B, both can pass in either direction depending on their relative concentration on either side of the membrane. More significantly, however, is the case in which the transporter is in fact an antiporter, where the transport of A in one direction is coupled to the transport of B in the other direction, whether B is measured or not. In radioisotopic assays for the uptake of A, B is usually unlabelled and hence unobserved. By contrast, so-called “untargeted” [255,256,257] mass spectrometric methods show clearly that there is a massive amount of efflux as well as influx when cells are exposed to new drug and nutrient sources [258]. In addition, the energy coupling elements of concentrative or efflux transporters are largely separate from those responsible for conducting the passage of the substrate through the membrane or can be made so [259].

“Multi-drug transporters” (MDTs) represent a particularly clear example of this; often, a transporter is labelled as an MDT involved in efflux because lowering its activity makes an organism more sensitive to a cytotoxic drug. However, in some cases (e.g., [260]), the removal of such a transporter actually makes the organism more sensitive to some other substances! Only “omics” methods in which many substances are measured simultaneously can easily disentangle this kind of behaviour.

### 1.9. Genome-Wide Analysis of Drug Uptake and Efflux in E. coli

**Figure 6:**
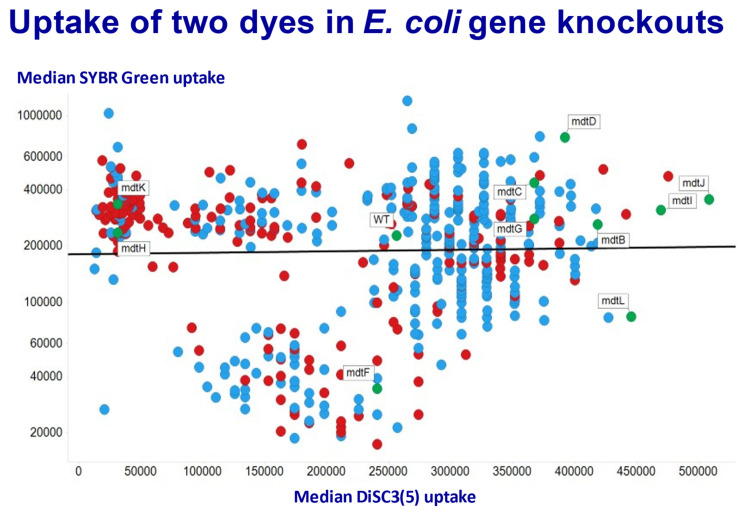
Dual roles of so-called multidrug transport proteins in *E. coli*. Data redrawn from those published with Open Access at [261]. Plotted are the median uptakes of SYBR Green and DiSC3(5) by various knockout strains relative to the Wild Type (WT). The ranges are, respectively, 70-fold and 36-fold. So-called y-genes (genes of nominally unknown function [278,279]) are encoded in red. Mdt gene knockouts are labelled (and have green symbols).

In recent work [261,262,263], we have recognised that the existence of genome-wide knockout (and overexpression) collections allows for the high-throughput assessment of the uptake of small molecule substrates. Since fluorophores are perfectly good surrogates for these purposes [264], and their uptake admits easy assessment using flow cytometry [265,266,267,268,269,270,271,272], we have been able to assess the influence of the expression of individual transporters on the uptake of various fluorophores. Figure 6 gives an example from *E. coli* [261], using a wild type and some 530 knockouts, of the effect of such knockouts on the steady-state uptake of two dyes, viz DiSC3(5), a carbocyanine dye responsive to membrane energisation [70], and SYBR Green, an intercalating dye whose fluorescence is massively enhanced upon binding to (especially double stranded [273]) DNA [274,275]. We here focus on the so-called multidrug transporter genes of the mdt family. Although the effect of knocking them out individually is, as expected [276], mainly (for mdt B,C,D,G,I, and J) to increase the steady-state uptake of DiSC3(5), that of mdtH and mdtK has the opposite effect, and uptake is very significantly decreased, indicating that normally these can act as influxers for this molecule. In a similar vein, the uptake of SYBR Green is lowered when mdtF and mdtL are knocked out.

While one can never exclude the contribution of pleiotropic effects (e.g., [277]), the obvious conclusion from these kinds of genome-wide study is that many individual transporters can potentially contribute to the uptake and efflux of any stated substrate of interest. Where this is the case, knocking a single one out may not have measurable effects, since the others can “take up the slack” [276]; this does not of course then mean that the gene knocked out did not catalyse the flux of the substrate of interest—absence of evidence is not evidence of absence.

A convenient method for invoking genome-wide diversity via gene disruption is the use of transposon insertion, but this has the disadvantage that the inactivation of essential genes in haploid organisms is missed. Thus, Webber and colleagues [280] have developed a system (TraDIS-Xpress) based on a transposon linked to an inducible promoter and used this to determine transporters (and other genes) involved in resistance to triclosan [280] and to fosfomycin [281]. This kind of strategy will be vital in further uncovering transporter-mediated contributions to antimicrobial resistance (AMR).

### 1.10. Recent Approaches to the De-Orphanisation of Mammalian “Orphan” Transporters

Some 1000 genes in the human genome encode transporters [7], of which the largest class, amounting to roughly half [282,283,284,285,286,287,288], is represented by the SoLute Carrier (SLC) class (http://slc.bioparadigms.org/, accessed on 15 September 2021). As noted above, even for those that have at least one known substrate, there are doubtless many more to be found. More significantly, at this stage of knowledge, many of them are complete “orphans” in that not a single substrate is in fact known. One recent example of de-orphanisation is that of the mitochondrial transporter SLC25A51, which turns out [108,289] to be an NAD^+^ importer. This de-orphanisation hinged upon a successful combination of genomics, metabolomics, CRISPR-Cas9-mediated gene editing, and genetics. Other strategies include binding assays [107], the use of direct assays in *Xenopus* oocytes [290], and others that are covered in a recent and comprehensive review [119].

### 1.11. Selectivity and Drug Targeting by the Use and Exploitation of Varying Expression Profiles

**Figure 7:**
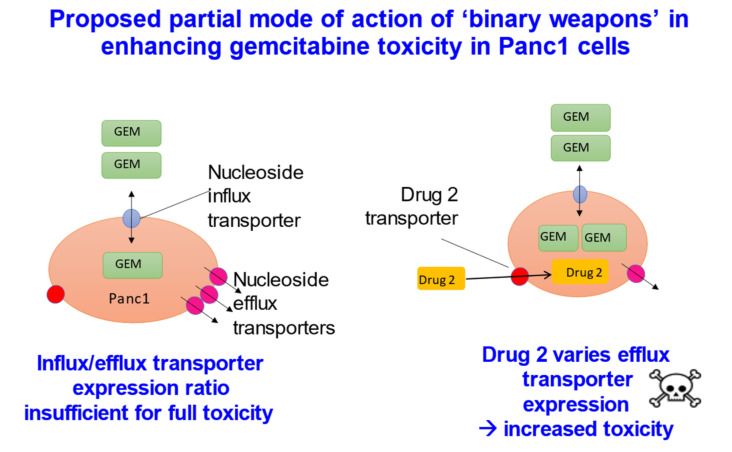
“Binary weapons” based on drug transporters [294]. The cytotoxic drug gemcitabine (GEM, a fluorinated cytosine nucleoside analogue) when added at a certain low concentration to Panc1 pancreatic cancer cells was barely cytotoxic (left panel). However, when a second drug was added, which was itself also non-toxic, the combination was substantially more toxic. What had occurred was that, in response to the GEM, the cells had increased the expression of the efflux transporter ABCC2 (MRP2) some 12-fold; the second drug inhibited this process, and in a cell-selective manner.

Given the need for transporters if drugs are to cross cell membranes, one can make a virtue of necessity [284] by seeking either to exploit their natural expression profiles [291,292,293] or to vary them explicitly [294], so as to target them to particular tissues [295]. The latter has obvious benefits in oncology [296,297,298,299,300], where the necessary cytotoxicity of many drugs, such as nucleoside analogues, is rather non-specific. The strategy can be used for modifying both influx and efflux transporters, though the latter is likely to prove more efficacious [276]. In one example [294] (Figure 7), the second drug in the “binary weapon” strategy decreased the expression of a relevant efflux transporter some 12-fold, in a cell-selective manner. This binary weapon strategy potentially holds much promise for targeting cytotoxic anti-cancer drugs.

**Figure 8:**
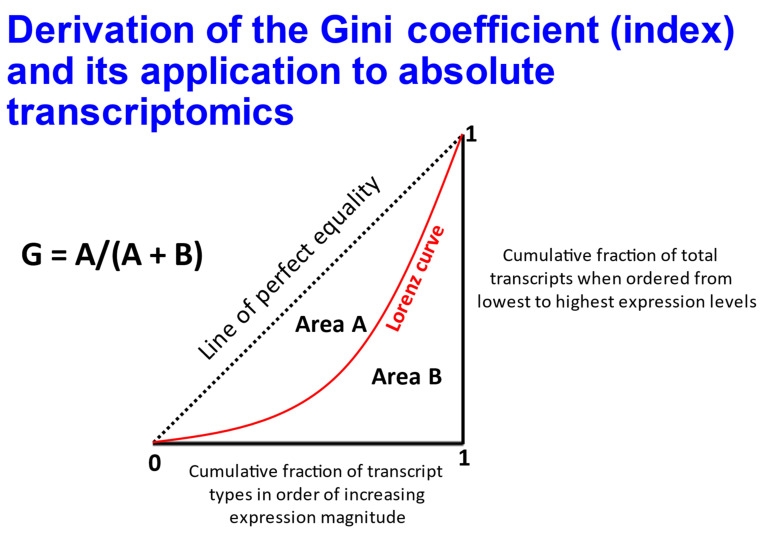
Illustration of the derivation of the non-parametric Gini coefficient for describing the inequality of a distribution (here the variation of transcript levels between cell lines). This was achieved by rank ordering the value of the different examples according to their expression levels, as indicated.

**Figure 9:**
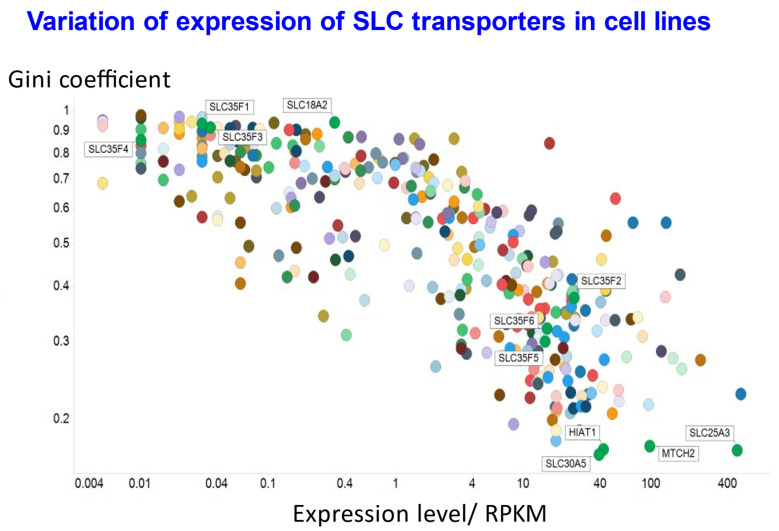
Variation of Gini coefficient and median expression for 410 SLC transporters in 56 cell lines. Data were obtained from previous open access publications [85,86] and a subset replotted. A few transporters are labelled to illustrate some of the SLCs with the lowest value of the Gini coefficient (lower right), one with a high value and a reasonable expression (SLC18A2), and the six members of the SLC35F family. The transcript expression levels are expressed through a widespread normalisation (see [305], but also [306,307]) as reads per kilobase million (RPKM).

The fact that expression levels of SLCs often vary considerably was illustrated by us previously [86], using a publicly available dataset [85]. The Gini coefficient (see [86,100,301,302]) (Figure 8) describes the heterogeneity of a distribution in a simple, non-parametric manner, and takes values between zero and 1. Some of the cell line data are replotted in Figure 9, where the Gini coefficient is plotted against their median expression level as assessed by RNASeq. Specifically, we use these data to illustrate four points: (i) the very fact that there are a great many uptake transporters (at least 400), that may or may not be expressed in different cells; (ii) those transporters with the lowest Gini coefficient, indicating their relative homogeneity of expression between cell lines (which may be referred to as GiniGenes [86,100]); (iii) an example (in the form of SLC18A2, a vesicular monoamine transporter [303]) of a transporter with both a high Gini coefficient, indicating a very restricted expression, and a reasonably high expression level; and (iv) the substantial variation in Gini coefficient for the six members of the SLC35F family, with three being quite selectively expressed (SLC35F1,3,4) while the others (SLC35F2,5,6) are expressed over a very broad range of values, as shown explicitly elsewhere for SLC35F2 [97]. Overall, 61 of the 410 transporters (15%) plotted in Figure 9 have a Gini coefficient over 0.8, 105 (26%) exceed 0.7 and 173 (42%) exceed 0.5. By contrast, the Gini coefficient for income inequality in different countries (its usual domain of application [304]) https://www.indexmundi.com/facts/indicators/SI.POV.GINI/rankings shows only 14 countries/159 (9%) with a Gini coefficient exceeding 0.5.

### 1.12. Transporters and Prodrugs

Another strategy that has been widely used to improve cellular drug uptake is the use of so-called prodrugs, in which a drug is modified by the addition of a moiety that, although without direct pharmacological activity at a target receptor, assists the passage of the drug to its target (e.g., [308,309,310,311,312,313,314,315,316,317,318]). These necessarily involve transporters, although their identity is not always known. Several recent examples showing high efficacy are based on the SLC7A5 (LAT1) transporter [319,320,321,322,323,324]. In one recent instance [321], the uptake of the anti-inflammatory salicylic acid was enhanced more than five-fold by fusing it with a phenylalanine moiety. Sometimes the prodrug is more lipophilic than its parent, and this is taken in some quarters to mean that it therefore must be passing through bilayers. As pointed out before [8], however, “in actual practice, the reformulation of a water soluble drug with lipidization modifications is difficult to execute successfully, and there is not a single example of a drug presently sold whereby medicinal chemistry was successfully used to convert a non-brain-penetrating drug into a molecule that crosses the BBB {blood–brain barrier} in pharmacologically significant amounts” [325].

### 1.13. Transporters and Adverse Drug Reactions

Notwithstanding all the benefits of small molecule drugs, it remains the case that they are also widely associated with various morbidities and mortalities, often referred to as “adverse drug reactions” (ADRs) (e.g., [326,327,328,329,330,331,332,333,334,335,336,337,338,339,340,341,342,343,344,345,346]). Despite the lengthy and complex regulatory hurdles that drugs must overcome before being marketed (and toxicity remains a major cause of so-called “attrition” where drug candidates are pulled before they even get to market [347,348,349,350,351,352,353]), ADRs are extremely common (and hard to anticipate, given the huge genetic and phenotypic variation in human populations [354]). Due to non-linearities in biochemical kinetics, averaging across many cells or tissues necessarily hides the true biology [267,355], and thus lumping heterogeneous cells into tissues will always miss such problems [2]. Our contention is that in many cases these ADRs are mediated via transporters, especially since when concentrative transporters can potentially cause massive accumulation in particular cells. Failure or inhibition of efflux transporters also has a major role to play in drug toxicity.

Consequently, the involvement of transporters in ADRs and drug cytotoxicity is well-established (see above and, e.g., [294,336,339,340,356,357,358,359,360,361,362,363,364,365,366,367,368,369,370,371,372,373,374,375,376,377,378,379,380,381,382,383,384,385,386,387,388,389,390,391,392,393,394]), providing further evidence for the major roles of transporters in both pharmacokinetics and pharmacodynamics.

### 1.14. Transporters, Antibiotics, and Antimicrobial Resistance (AMR)

Antimicrobial resistance (AMR) is a major human health problem (e.g., [272,395,396,397,398,399,400,401,402,403,404,405,406,407,408,409,410,411,412,413,414,415,416,417,418,419]). Most pertinently, efflux transporters are well-recognised as a major source of AMR (e.g., [276,397,420,421,422,423,424,425,426,427,428,429,430,431,432,433,434,435,436,437,438,439,440,441,442,443,444,445,446,447,448,449,450,451,452,453,454,455,456,457]). The outer membrane also contributes significantly to the permeability barrier in Gram-negatives (e.g., [265,434,443,444,448,458,459,460,461,462,463,464,465,466]). However, another specific area in which the role of transporters is largely unrecognised—albeit this is a specific subset of drug transport—pertains to the uptake transport of anti-infectives to their sites of action [439,467]. This can involve both the targets within the microbe and the host’s transporters when (as is common, e.g., [468,469,470,471,472,473,474,475,476,477,478,479,480,481,482,483,484,485]) the infective agents reside intracellularly. A particularly clear example is given by *Mycobacterium tuberculosis*, the causative agent of TB, where the very striking lack of correlation between in vivo and in vitro drug potencies is easily and necessarily explained via transporter activities [139,151,486,487,488]. Many orally prescribed antibiotics enter the host via SLC15 family members [489,490], while some of the relatively few known microbial uptake transporters for anti-infectives are listed in Table 1. What evidence there is implies that there are multiple means of uptake, which is why identifying individual transporters for successful antibiotics has proven difficult [276]. On the flipside, of course, when we recognise the relevant transporters and/or their structure–activity relationships governing cell permeability, we can exploit them [437,444,491,492,493,494,495].

### 1.15. Molecular Dynamics of Transporter Reactions

Given, as mentioned, the present impossibility of detecting the molecular pathway of drug transport directly, one alternative is to calculate it from first principles, which for these purposes means via the use of molecular dynamics (MD). MD allows the calculation “ab initio” of the molecular motions of molecules during their normal activity. Although computationally demanding (something that becomes much less of an issue over time, e.g., [512,513]), it is perfectly suited to calculating the mechanisms of substrates transport across membranes [514]. We have discussed this in more detail elsewhere [2], and so we simply include here some of the more recent developments. Thus, Jia et al. [515] could mirror precisely the experimental findings underpinning the behaviours of a xylose transporter. Other findings uncovered an electrostatic lock in emrE [516] (see also [517,518]), established the molecular basis of sodium-coupled transporters [519], and illustrated the mechanism of transporters as varied as acrB [520,521,522], the vitamin B_12_ importer BtuCD [523], the maltose transporter ATPase MalK2 [524], McjD (an antibacterial peptide ABC transporter from *E. coli*) [525], proton oligopeptide transporters [526], hexameric urea transporter UreI from *Helicobacter pylori* [527], and the mammalian transporters SLC1A1 (excitatory amino acid transporter EAAT3) [528], SLC2A1 (glucose transporter GLUT1) [529], SLC4A1 (“band 3” protein, bicarbonate/anion exchanger) [530], SLC6A4 [531], SLC7A10 [532]. By contrast, studies of membrane-embedded molecules such as aquaporins, when conducted with high protein concentrations resembling those in biomembranes [533,534,535,536], show that even water molecules do not pass through the bilayer (see a direct illustration at http://www3.mpibpc.mpg.de/groups/de_groot/gallery/aqp1_snapshot.jpg/). In several cases, the MD simulations are accompanied by confirmatory X-ray structures (e.g., [530,535,536]).

### 1.16. Uptake Transporters as Drug Targets

Although our chief interest here relates mainly to the role of solute carriers in drug disposition, we would be remiss not to mention that, largely because they have been seriously understudied [288], SLCs themselves necessarily constitute potentially valuable and novel drug targets [11,286,537,538,539,540,541,542,543,544,545,546,547,548,549,550,551,552]. It is reasonable that the technical improvements in cryo-EM will contribute to the rational design of such drugs [553], as well as the many other activities ongoing (e.g., as summarised in [131]).

### 1.17. What Are the “Real” Substrates of Drug Uptake Transporters?

While some drugs that are SLC substrates are, or are semi-synthetic analogues of, natural products, most modern drugs are entirely synthetic in nature, and so natural evolution had no known exposure to them. It is then at least reasonable to enquire as to what the “normal” substrates of these molecules are that happen also to allow them to transport drugs. The principle of molecular similarity (e.g., [201,554,555,556,557,558,559,560,561,562,563,564]) suggests that molecules that have similar structures should tend to have similar activities, so the question then becomes “to which molecules are marketed drugs most similar”? This is a cheminformatics question, and the answer depends in part on the nature of the structural encoding, although most encodings of “actually” similar molecules show a Tanimoto similarity exceeding 0.8 or so, a number that may be used as a kind of benchmark. Our initial assumption was that successful, marketed drug should bear structural similarities to endogenous human metabolites [565,566,567,568], which have been catalogued in metabolic reconstructions [569] and elsewhere (e.g., [570]). However, this accounts for only a small percentage (~15% [83,200,568]), and the true answer—possibly unsurprisingly, post hoc—is that most drugs actually bear similarities to natural products, whose uptake via SLCs may be assumed to be, or to have been during natural selection, of some nutritional or medical benefit to the host [200,264]. This also brings to the fore the important role of natural products in drug discovery [9,571,572,573,574,575,576,577,578,579,580,581,582,583,584,585,586]. Natural products are, of course, famous for breaking [587,588,589,590] many of the “rule of 5” [591] guidelines, and include many very clear examples of drugs that cannot possibly diffuse through lipid bilayers in intact biological cells.

### 1.18. Why Do SOME Solvents Increase the Rate of Drug Uptake?

Anecdotally, there is a common assumption that because solvents such as DMSO can increase the rate of drug uptake they must be doing so by solubilising the drug, or at least by assisting its solubilisation, in the phospholipid bilayer part of biomembranes. This would then be seen as some kind of evidence for the importance of bilayer transport, but in fact this does not follow at all. The issue with insoluble drugs is that transporters require their substrates to be bound, and that this normally happens via solubilisation in the aqueous phase. Rocks and crystals and amorphous solids are not direct substrates of drug transporters; molecules are. All that solvents such as DMSO are then doing is increasing the rate of solubilisation of drug solids and their presentation to the transporters as single molecules (the necessarily preferred substrates) in solution.

## 2. Discussion

Real biological membranes possess a high protein:lipid ratio, often as much as 3:1 by mass. Consequently, they do not remotely behave in a manner similar to pure, artificial, phospholipid bilayers. Additionally, the transport of small organic molecules (including drugs) across them commonly requires the intercession of proteinaceous transporters (e.g., [2,3,6,8,101,287,370,592,593,594,595,596,597,598,599,600,601,602,603,604,605,606,607,608,609,610,611,612,613,614,615,616,617,618,619,620]), and there is in fact no actual evidence whatsoever for any significant flux across native, undamaged biomembranes through whatever phospholipid bilayer may be present. This contrasts with a widely held set of assumptions, based in part (it is assumed) on what can be observed in pure phospholipid bilayers that admit the transport of all kinds of small molecules, albeit through transient aqueous pores.

Identifying these transporters of small molecules can be performed (e.g., [96,97,99,101,171]) by manipulating their expression, including under conditions in which their substrates are otherwise toxic. The idea is that removing (or otherwise inhibiting) a transporter that normally helps a toxic drug to enter a cell should increase the host’s resistance to it, while overexpressing the transporter would make the cells more sensitive. More generally, understanding the co-variation between the uptake of a molecule and the expression of its potential transporters gives a strong indication of which they are.

The recognition that in real biological membranes the transbilayer transport through phospholipids of small molecule drugs, nutrients, and biotechnology products is negligible also explains straightforwardly the following well-established facts:

- The negligible uptake of drugs and substrates in some tissues, including via the blood–brain barrier (and equivalents in the retina, testes, and other tissues), where relevant transporters are absent;
- The extreme heterogeneity of uptake of a given molecule in different organs, tissues, and organisms despite little substantive variation in their lipid physical properties;
- The existence of transporters for all kinds of small molecules (even water, acetate, ammonia, glycerol, etc., as well as entirely hydrophobic molecules such as alkanes [621,622,623]) that had previously been assumed to lack them;
- A variety of cases in which individual defined transporters can be shown to account for the overwhelming bulk of measured fluxes;
- The need for such transporters in order to effect drug uptake, mirroring the widespread recognition that they can serve to efflux them (and thereby created resistance to their activity);
- The role of transporters in drug-mediated toxicity (e.g., [366,624]);
- The poor correlation between the uptake of small molecules and simple physicochemical properties such as log P or log D (many examples, such as those in [2,6,83,625,626,627]).

## 3. Looking to the Future

The two chief questions posed earlier (“what are the substrates for a given trasporter?” and “what are the transporters for a given substrate?”) are normally addressed experimentally, using a variety of the methods described above. As intimated above, the exponential increase in computer power will eventually allow the methods of molecular dynamics to admit these “measurements” entirely by calculations based on simple force fields, *de novo*. In addition, given the success of so-called deep learning [628,629,630] methods in predicting the structures of proteins [631,632,633,634,635,636,637,638,639,640,641,642,643,644,645], novel receptor–ligand interactions [646,647,648,649,650], and a variety of other protein and small molecule properties (e.g., [201,651,652,653,654,655,656,657,658,659,660,661,662,663,664,665]), it seems likely that we shall soon have available in silico methods for predicting transporter substrates directly from protein sequences, and the likeliest transporters from candidate substrate structures of interest.

## 4. Conclusions

On the basis of the present evidence, the transport of drugs and nutrients through phospholipid bilayers in real biomembranes is negligible. Progress in understanding drug distribution profiles is thus to be made by establishing which transporters they use, the expression profiles of those transporters, and the kinetic rate equations that they obey. Armed with these it will be possible to model, to analyse, to understand, and to exploit our principled knowledge of drug distributions both mechanistically and with confidence.

## Figures and Tables

**Table 1:** Some examples of differential resistance to anti-microbial drugs involving uptake transporters.

Antibiotic	Transporter	Comments	Selected Reference(s)
Aminoglycosides			[496]
Chloramphenicol	YdgR	*E. coli*. Proton-dependent oligopeptide transporter analogue	[497]
Cycloserine			[498]
5-fluocytosine	FCY2	Various *Candida* spp.	[499,500]
Fosfomycin			[501,502]
Pacidamycin	Opp PA14	*Pseudomonas aeruginosa*	[503] [504]
Pentamidine	Three adenosine-based transporters		[441,505]
Quinoline antimalarials	AAT1		[506]
Reviews			[441,507,508,509]
Tetracyclines	Two unknown transporters		[510,511]

## Data Availability

Not applicable.

## Sample Availability

Samples of the compounds are not available from the authors.
